# The first dataset of de novo transcriptome assembly of *Heterotrigona itama* (Apidae, Meliponinae) queen larva

**DOI:** 10.1016/j.dib.2020.105235

**Published:** 2020-02-01

**Authors:** Amin Asyraf Tamizi, Nazrul Hisham Nazaruddin, Wee Chien Yeong, Muhammad Faris Mohd Radzi, Mohd Azwan Jaafar, Rogayah Sekeli

**Affiliations:** Agri-Omics and Bioinformatics Programme, Biotechnology and Nanotechnology Research Centre, Malaysian Agricultural Research and Development Institute (MARDI), 43400 Serdang, Selangor, Malaysia

**Keywords:** Caste, Entomology, Meliponine, RNA-seq, Transcriptomics

## Abstract

*Heterotrigona itama* is a species of stingless bee recently domesticated (or reared) for honey production in a few Southeast Asian countries namely Malaysia and Indonesia. Being categorized in the clade Corbiculata together with the honeybees (*Apis* spp.) and bumble bees (*Bombus* spp.), the stingless bees are highly social in which the colony members are subjected to labor division where a queen functions as the reproductive caste. In this data article, we provide a resource encompassing a transcriptome profile (de novo assembled) from *H. itama* queen larva – the first report of transcriptome assembly for this species. The generated data is pivotal for the characterization of important genes and biological pathways in order to further improve our understanding on the developmental biology, behavior, social structure and ecological needs of this eusocial hymenopteran insect from the molecular aspect. The raw RNA sequencing data is available at NCBI Sequence Read Archive (SAR) under the accession number SRP230250 and the assembled reads are deposited at DDBJ/EMBL/Genbank as Transcriptome Shotgun Assembly (TSA) under the accession GIIH00000000.

**Table:** Specifications

Subject Area	Entomology
Specific subject area	Transcriptomics
Type of data	RNA-seq data (paired-end) and assembly of reads
Method for data acquisition	Illumina HiSeq™ 3000 sequencing platform
Data format	Raw sequence reads (FASTQ) and assembled contigs (FASTA)
Experimental factors	A single queen larva (3rd instar; feeding stage) was harvested from an active colony placed close to a forest reserve at MARDI, Serdang.
Experimental features	The hive dedicated for supplying biological materials was reared near a forest reserve full of vegetations and botanical resources for the colony. The larva was harvested during sunny season in September 2018. Total RNA was extracted and purified from whole larva using an optimized protocol and sent for sequencing.
Data source location	Malaysian Agricultural Research and Development Institute (MARDI), 43400 Serdang, Selangor, Malaysia.
Data accessibility	The raw sequence reads can be obtained through NCBI SRA accession number SRP230250 (https://www.ncbi.nlm.nih.gov/sra/SRP230250) and the assembly data has been deposited in the NCBI TSA with accession number GIIH00000000 (https://www.ncbi.nlm.nih.gov/nuccore/GIIH00000000).

**Table:** 

**Value of the data** • The data set is the first deposited source of RNA-seq and assembled transcriptome from a female larva of Malaysian stingless bee (*Heterotrigona itama*). • The dataset provides starting evidence and is useful as a reference to the scientific research community interested in studying biological development or growth of Malaysian stingless bees especially for species with no reference genome. • The expression profiles are available as raw sequence reads that can be further processed and analyzed by researchers according to their preferences and parameters.

## 1 Data

The dataset contains raw sequencing data obtained through the transcriptome sequencing of a female *H. itama* larva from the queen caste. A queen larva at feeding stage (3rd instar) was collected from a mother colony and snap-frozen in liquid nitrogen (N_2_), and high quality total RNA was extracted before sent for sequencing through paired-end Illumina sequencing technology. De novo assembly was performed using Trinity and contigs were annotated against seven databases using multiple software. An overview of the data and sequencing assembly of *H. itama* data is presented in Table 1 and Table 2. The raw data file (reads in FASTQ format) was deposited at NCBI SRA database under accession no. SRP230250 and the transcriptome assembly data was deposited at NCBI TSA with accession number GIIH00000000.

**Table 1:** Data production of RNA-seq.

Sample	Raw Reads	Clean reads	Clean bases	Error (%)	Q20 (%)	Q30 (%)	GC (%)
Queen larva (3rd instar)	102300462	99370410	14.9G	0.01	97.48	93.50	42.18

Q20: percentages of bases whose correct base recognition rates are greater than 99% in total bases.

Q30: percentages of bases whose correct base recognition rates are greater than 99.9% in total bases.

**Table 2:** Overview of transcripts and unigenes assembled using Trinity (version r2014-04-13p1).

Attributes	Transcripts	Unigenes
Min. length	201	201
Max length	58291	58291
Mean length	1837	1837
Median length	904	904
N50	3588	3588
N90	717	718
Total nucleotides	101280555	101277047
**Total number**	**55148**	**55135**

## 2 Experimental design, material and methods

### 2.1 Insect material and RNA extraction

The queen larva, whose species had been validated through DNA barcode (cytochrome oxidase subunit I), was picked from a very healthy colony that had been placed at a dedicated research plot adjacent to a secondary forest for more than 6 months. Larva instar 3 (feeding stage) was selected for RNA sequencing since this stage was reported to be the ‘deciding stage’ for caste differentiation in female stingless bee larvae [1]. Total RNA was extracted from whole larva using Aurum™ Total RNA Mini Kit (Bio-Rad) according to the manufacture's protocol. Each RNA sample was extracted from single insect in order to prevent sequence noise.

### 2.2 RNA preparation and sequencing

Prior to Complementary DNA (cDNA) libraries preparation and sequencing, quality check (QC) of the total RNA was done as follows: preliminary quantitation (Nanodrop), degradation and contamination tests through agarose gel electrophoresis, and final integrity and quantitation tests (Agilent 2100). The RNA was then processed following these steps: Enrichment of mRNA using oligo(dT) beads, removal of rRNA using a specialized kit and fragmentation of mRNA. Afterward, the cDNA was synthesized from the mRNA fragments using random hexamers and reverse transcriptase. Following the first-strand synthesis, a custom second-strand synthesis buffer (Illumina) is added together with dNTPs, RNase H and *Escherichia coli* polymerase I to generate the second strand by nick-translation followed by two rounds of cDNA purification using AMPure XP beads. The cDNA was then proceeded for terminal repair, A-tailing, ligation of sequencing adapters, size selection and PCR enrichment. For library quality assessment, the cDNA library concentration was determined using Qubit 2.0 fluorometer (Life Technologies), and the insert size was checked on Agilent 2100 and quantified to greater accuracy by quantitative PCR (qPCR) (library activity >2 nM). Finally, the prepared cDNA libraries were fed into Illumina machines according to activity and expected data volume.

### 2.3 RNA-seq data analysis (raw reads handling and de novo assembly) and gene annotation

The raw data from Illumina was transformed to Sequence Reads by base calling and recorded in a FASTQ file. Raw reads were cleaned/filtered as follows: (1) removing reads with adaptor contamination, (2) removing reads when uncertain nucleotides constitute more than 10% of either read (N > 10%) and (3) removing reads when low quality nucleotides (base quality less than 20) constitute more than 50% of the read. De novo transcriptome reconstruction was carried out using Trinity (version r2014-04-13p1) with a minimum read length of 200 and k-mer = 25. The Trinity workflow followed the Inchworm, Chrysalis and Butterfly modules [2]. The summary of sequencing and assembly data are tabulated in Table 1, Table 2. The contigs were then clustered with Corset [3] to remove redundancy.

**Fig. 1:**
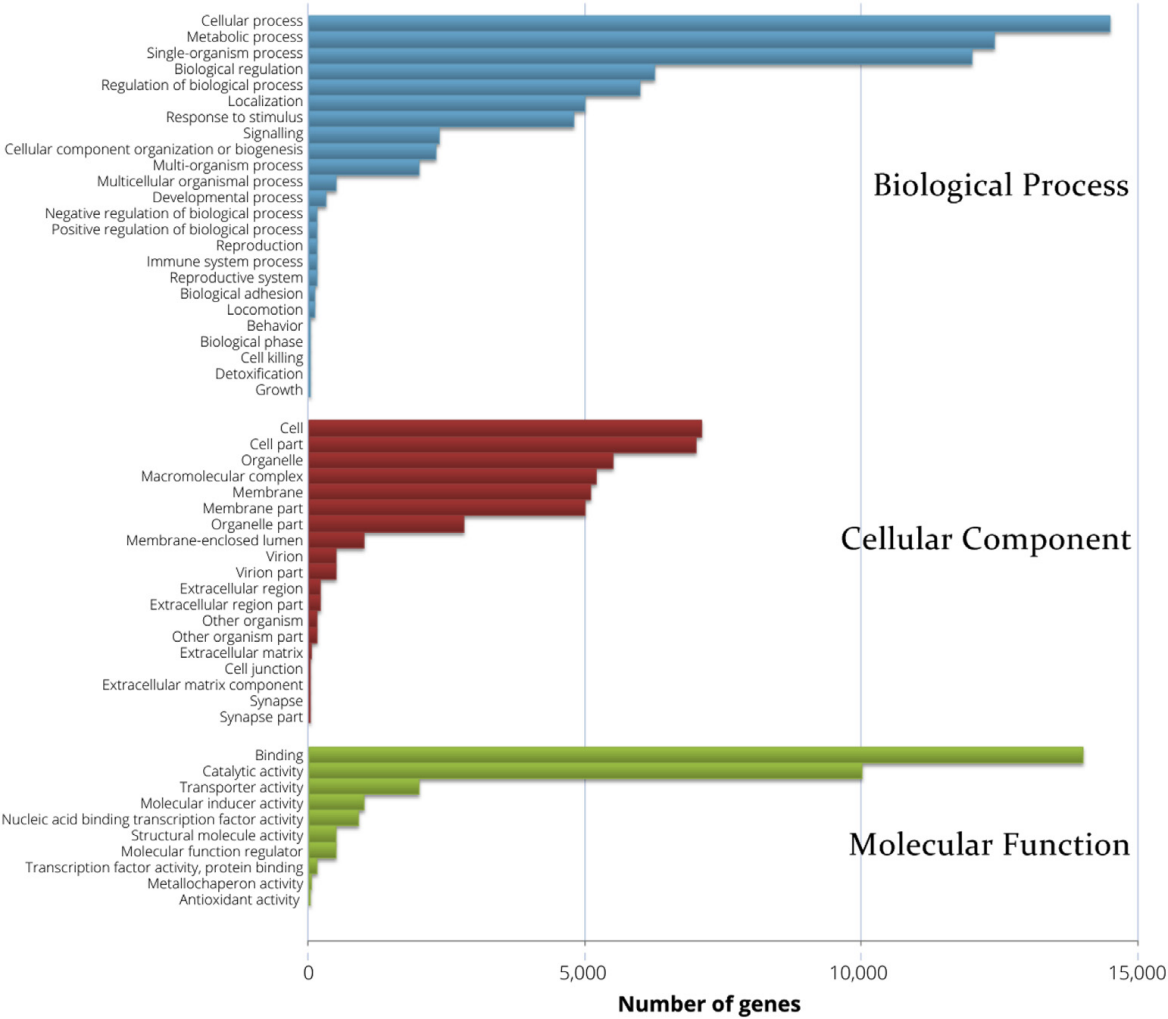
GO classification distribution of annotated genes from *H. itama* queen larva.

Gene functional annotations were carried out using Diamond (v0.8.22), KAAS (r14 0224), NCBI Blast (v2.2.28+), hmmscan (HMMER 3) and blast2go (b2g4 pipe_v2.5) software. A total of seven databases including Nr, Nt, KO, Swiss-Prot, Pfam, GO (Fig. 1) and KOG were used to annotate the contigs and 55,135 of unigenes had been successfully annotated (Table 3).

**Table 3:** The statistics of annotated genes (unigenes) by different databases.

Database	Number of unigenes	Percentage (%)
Nr (NCBI non-redundant protein sequences)	24171	43.83
Nt (NCBI nucleotide sequences)	30532	55.37
KO (KEGG Orthology)	12012	21.78
Swiss-Prot	19214	34.84
Pfam (Protein family)	22200	40.26
GO (Gene Ontology)	22216	40.29
KOG (euKaryotic Orthologous Groups)	14371	26.06
Annotated in all databases	8526	15.46
Annotated in at least one database	34787	63.09
**Total Unigenes**	**55135**	**100.00**
